# Co-production of amino acid-rich xylooligosaccharide and single-cell protein from paper mulberry by autohydrolysis and fermentation technologies

**DOI:** 10.1186/s13068-021-02095-6

**Published:** 2022-01-07

**Authors:** Yang Gu, Yingying Hu, Caoxing Huang, Chenhuan Lai, Zhe Ling, Qiang Yong

**Affiliations:** 1 Jiangsu Co-Innovation Center of Efficient Processing and Utilization of Forest Resources, College of Chemical Engineering, Nanjing Forestry University, Nanjing, 210037 China; 2 State Key Laboratory of Pulp Paper Engineering, South China University of Technology, Guangzhou, 510640 China

**Keywords:** Paper mulberry, Autohydrolysis, Xylooligosaccharide, Amino acids, Single-cell protein

## Abstract

**Background:**

Autohydrolysis is an extensively investigated pretreatment method due to its environmental friendliness. During autohydrolysis, most xylan from hemicellulose can be converted into xylooligosaccharides (XOS), and cellulose in the autohydrolyzed residues can be transformed into glucose after enzymatic hydrolysis. Both of these are value-added biochemicals in the biorefining process. In this work, paper mulberry (PM), which contains abundant protein, was utilized as a raw material to coproduce XOS and single-cell protein (SCP) through autohydrolysis and fermentation technologies.

**Results:**

The results showed that 8.3 g of XOS and 1.8 g of amino acids could be recovered in the autohydrolysate (based on 100 g raw material) after autohydrolysis (170 °C, 1 h). Moreover, 5.7 g of low-DP XOS along with 1.8 g of amino acids could be further obtained from the autohydrolysate after hydrolysis with endo-β-1-4-xylanase. In addition, 20.1 g of fermentable monosaccharides was recovered after hydrolyzing the autohydrolyzed PM with cellulase, which can be used to produce 4.8 g of SCP after fermentation with *Candida utilis*.

**Conclusion:**

As a valuable application of PM, a novel process is proposed to coproduce amino acid-rich XOS and SCP through autohydrolysis. The carbohydrate of PM is effectively converted to high value-added products.

## Background

Paper mulberry (*Broussonetia papyrifera,* PM), a hardwood from the *Moraceae* family, is widely distributed in East and South Asia and exhibits fast growth, ease of breeding, strong tillering ability, and pruning resistance [1, 2]. In East Asia, the plant cortex of PM is widely used as a raw material for paper manufacture [3], and the fruit from PM is used as a traditional medicinal for its pharmacological effects such as antioxidant [4], anti-inflammatory [4], and immunomodulation activities [2], and as a treatment for Alzheimer’s disease [5]. Additionally, the leaves of PM are ensilaged for livestock farming due to their rich dietary fiber. However, the branches and twigs remain unutilized components of PM. Interestingly, these branches and twigs contain not only cellulose, hemicellulose and lignin as regular lignocellulosic biomasses but also abundant protein. Hence, the branches and twigs of PM are attractive substrates for the biorefinery process to obtain hemicellulose and cellulose as high-value products as well as providing other biomass [6]. However, it is difficult to effectively separate hemicellulose from cellulose due to their compact structure. Therefore, an appropriate pretreatment method is necessary for the coproduction of amino acid-rich XOS and fermentable monosaccharides from PM. Through pretreatment, most of the xylan in the hemicellulose can be converted into value-added chemicals such as xylooligosaccharides (XOS) and xylose, and the cellulose can be enzymatically hydrolyzed into glucose for further fermentation [7]. Meanwhile, the protein can be degraded into amino acids. Therefore, the pretreatment method is the key to the efficient utilization of PM.

Among all pretreatment technologies, autohydrolysis is considered an economical, ecofriendly, and concise pretreatment method, because it is a reagent-free treatment that relies only upon temperature and pressure to both deacetylate xylan and increase the efficiency of enzymatic hydrolysis [8]. Moreover, XOS, as one of the value-added biochemicals depolymerized from xylan, is the main product of lignocellulose autohydrolysis [9]. Furthermore, in terms of the autohydrolysis of protein-rich lignocellulose, solubilized xylan can be obtained mainly in the form of XOS, and the protein can be degraded into amino acids that can be further purified for valuable prebiotic applications [10]. Hence, autohydrolysis was used in this work to coproduce amino acid-rich XOS and fermentable monosaccharides from PM.

XOS, a type of functional oligosaccharide generated from hemicelluloses during autohydrolysis, are beneficial feed additives in human and animal diets [11]. Chemically, XOS are oligosaccharides made of 2–10 xylose molecules connected by β-(1, 4) linkages [9]. XOS with low degrees of polymerization (DP ≤ 6), especially xylobiose and xylotriose, are particularly beneficial, because they are fermented in the hindgut and provide prebiotic effects to the consumer via selective stimulation of beneficial bacteria such as *Bifidobacteria* or *Lactobacilli*. Furthermore, research has shown that XOS can help reduce populations of pathogenic bacteria such as *Escherichia coli* [12, 13]. Based on these benefits, the demand for XOS has increased since the turn of the century. On the other hand, amino acids can be obtained as byproducts after autohydrolysis. Like XOS, amino acids are high-value biochemicals that can be added to the diets of humans and animals, especially essential amino acids. Essential amino acids cannot be synthesized in the human or animal body and must be ingested from food [14]. Hence, the XOS and the amino acids recovered in the autohydrolysate can be purified for further applications. Moreover, the autohydrolyzed PM can be enzymatically hydrolyzed by cellulase to produce fermentable monosaccharides.

Additionally, the fermentable monosaccharides obtained from enzymatic hydrolysis residues can be utilized for microbial fermentation. One of the topical fermentation products is single-cell protein (SCP). As a value-added biochemical, SCP is widely used in the agricultural field. SCP refers to the protein derived from cells of microorganisms grown on various carbon sources during fermentation [15]. Due to its high protein content, SCP is also called a bioprotein or microbial protein and has the potential to serve as an alternative to plant protein sources [16]. Compared with plant protein sources, SCP production is not subject to seasonality, nor does it rely on large areas of land and abundant water supply. SCP can be produced by various kinds of microorganisms, including yeast (e.g., *Candida utilis*), bacteria (e.g., *Cupravidus nectar*), and algae (e.g., *Chlorella vulgaris*) [16, 17]. Among those, yeast is the best choice because of its high protein content, rapid growth rate, lack of pathogenicity, fast digestibility, and overall palatability [18]. *Candida utilis* is a typical yeast strain for producing SCP, and it can utilize agricultural residues, industrial waste and other materials as substrates [19–21]. Therefore, enzymatic hydrolysates rich in fermentable monosaccharides from biorefinery processing are an ideal substrate to produce SCP.

In this work, a process was investigated for the coproduction of amino acid-rich XOS and SCP through autohydrolysis by utilizing the branches and twigs of PM. After autohydrolysis, hemicellulose was depolymerized into XOS, while the protein was hydrolyzed into amino acids. Afterward, the autohydrolysate and autohydrolyzed PM were hydrolyzed by endo-β-1–4-xylanase and cellulase to obtain low-DP XOS (degree of polymerization ≤ 6) and fermentable monosaccharides. *Candida utilis* was used to convert the fermentable monosaccharides into SCP. The obtained low-DP XOS, amino acids and SCP can be utilized for further applications.

## Results and discussion

### Compositional analysis of autohydrolyzed PM

In this work, the autohydrolysis pretreatment was carried out at 150 °C–190 °C for 1 h to evaluate how PM responded to the increase in autohydrolysis severity. The chemical compositions of autohydrolyzed PM, the recovery yield of glucan, and the removal yields of xylan, lignin and crude protein are shown in Table 1.

**Table 1:** Effect of reaction temperature on autohydrolyzed PM

Biomass	Glucan (%)	Xylan (%)	Lignin (%)	Crude Protein (%)	Recovery Yield (%)		Removal Yield (%)		Degradation Yield (%)
					Solid	Glucan	Xylan	Lignin	Protein
Raw material	39.4 ± 0.5	22.1 ± 0.3	24.9 ± 0.0	6.58 ± 0.0					
150 °C	42.5 ± 0.5	22.3 ± 0.3	23.3 ± 0.7	2.4 ± 0.0	91.7	98.9	7.5	14.3	67.1
160 °C	45.1 ± 0.3	18.4 ± 0.3	26.5 ± 0.5	2.2 ± 0.1	81.4	93.1	32.0	13.4	73.0
170 °C	49.6 ± 0.2	13.9 ± 0.2	28.7 ± 0.1	1.9 ± 0.0	72.4	91.1	54.4	16.7	79.5
180 °C	53.0 ± 0.6	9.1 ± 0.2	33.9 ± 0.1	1.5 ± 0.1	67.4	90.6	72.2	8.2	84.2
190 °C	53.3 ± 0.3	5.9 ± 0.1	38.7 ± 0.1	1.3 ± 0.0	63.7	86.2	83.1	1.1	87.4

According to Table 1, the major components of PM are 39.4% glucan, 22.1% xylan, 24.9% lignin (acid-insoluble + acid-soluble), 6.6% crude protein and 4.3% extractive. It was found that increasing the autohydrolysis pretreatment contributed to a lower solid recovery yield. Specifically, the solid recovery yield decreased from 91.7 to 63.7% as the reaction temperature increased from 150 to 190 °C. Moreover, the glucan recovery yield decreased from 98.9 to 86.2%, while the xylan removal yield increased from 7.5 to 83.1%. The glucan yield and xylan yield are consistent with those of other hardwood, such as tulip trees [22]. The reason for the results was that the autohydrolysis pretreatment leads to hydrolysis of most hemicelluloses along with a small amount of cellulose [23]. For lignin, the degree of delignification increased from 14.3 to 16.7% when the temperature was increased from 150 to 170 °C. However, when the temperature was increased from 170 to 190 °C, the removal yield decreased from 16.7 to 1.1%. This result can be attributed to the newly formed pseudolignin, which is derived from the byproducts from cellulose and hemicellulose [22, 24]. Only 16.7% of the original lignin was removed at 170 °C, suggesting that the autohydrolysis pretreatment results in the dissolution of a small portion of lignin. Sun et al. reported that 26% of the original lignin in *eucalyptus* was removed by autohydrolysis at 180 °C for 15 min, and Batalha et al. reported that 50% of the original lignin in sugarcane bagasse was removed by autohydrolysis at 180 °C for 40 min [25, 26]. The types of lignocellulosic materials respond differently to delignification under autohydrolysis conditions.

In addition, the crude protein content in PM was 6.6%. Compared with other lignocellulosic biomasses, such as poplar (0.03%) and larch (0.61%), PM is abundant in protein, which makes it a promising resource for amino acid production. The protein in PM can be degraded into amino acids during autohydrolysis pretreatment. As shown in Table 1, the degradation yield of protein increased from 67.1 to 87.4% when the temperature was increased from 150 to 190 °C. The increased yield can be attributed to the enhanced breakage of peptide bonds in protein at high temperature during autohydrolysis.

### Compositional analysis of autohydrolysate

Many lignocellulosic biomasses, such as corncob and poplar, can be used to produce XOS through autohydrolysis. In contrast to conventional lignocellulosic biomasses, PM is rich in protein, which can be degraded into amino acids during the autohydrolysis process. Additionally, most hemicelluloses were hydrolyzed, with an XOS fraction arising from this degradation [9]. To examine the effects of autohydrolysis pretreatment on XOS and amino acid production, the chemical composition of autohydrolysate was analyzed and is shown in Tables 2 and 3.

**Table 2:** Effect of reaction temperature on autohydrolysate

Biomass	X2-X6/(g/L)					X2-X6 (g/L)	XOS (DP > 6) (g/L)	Total XOS (g/L)	Amino acid (g/L)	Protein (g/L)	X2-X6 Yield (%)	Total XOS Yield (%)	Amino acid Yield (%)
	**X**_**2**_	**X**_**3**_	**X**_**4**_	**X**_**5**_	**X**_**6**_								
150 °C	0.0	0.2	0.0	0.1	0.0	0.3	0.0	0.3	2.3	0.5	1.0	1.2	28.5
160 °C	0.1	0.2	0.2	0.1	0.2	0.8	4.4	5.2	2.1	0.5	2.5	17.4	26.0
170 °C	0.4	0.6	0.9	0.7	0.6	3.1	8.5	11.6	2.4	0.5	10.5	37.4	28.8
180 °C	1.4	1.3	1.3	0.8	0.5	5.3	2.7	8.0	2.4	0.5	17.8	27.1	29.1
190 °C	0.4	0.3	0.1	0.0	0.0	0.7	0.7	1.4	2.6	0.6	2.4	4.8	32.6

**Table 3:** The types and amounts of amino acid in autohydrolysate

Temperature	150 °C	160 °C	170 °C	180 °C	190 °C
Thr (g/L)	0.13	0.13	0.10	0.11	0.13
Val (g/L)	0.11	0.10	0.13	0.16	0.14
Met (g/L)	0.04	0.04	0.04	0.06	0.08
Ile (g/L)	0.12	0.10	0.12	0.12	0.12
Leu (g/L)	0.21	0.15	0.20	0.20	0.25
Phe (g/L)	0.13	0.13	0.11	0.12	0.14
Lys (g/L)	0.22	0.13	0.16	0.17	0.17
His (g/L)	0.07	0.07	0.06	0.07	0.08
Arg (g/L)	0.16	0.16	0.15	0.17	0.17
Asp (g/L)	0.18	0.14	0.15	0.18	0.25
Ser (g/L)	0.10	0.13	0.13	0.13	0.13
Glu (g/L)	0.40	0.35	0.44	0.38	0.40
Pro (g/L)	0.08	0.10	0.11	0.08	0.08
Gly (g/L)	0.12	0.14	0.15	0.14	0.14
Ala (g/L)	0.13	0.10	0.16	0.12	0.13
Cys (g/L)	0.01	0.01	0.02	0.01	0.03
Tyr (g/L)	0.12	0.12	0.13	0.13	0.14
∑(g/L)	2.32	2.12	2.35	2.38	2.58

As shown in Table 2, the concentration of total XOS in the autohydrolysate displayed an increasing trend, and subsequently decreased with increasing reaction temperature. The maximum total XOS concentration was 11.0 g/L and was achieved at 170 °C. However, when the temperature was over 170 °C, the total XOS yield declined, because the original xylan was depolymerized into xylose or excessively degraded to dehydration products. The highest total XOS yield of 37.4% (170 °C,1 h) was in good agreement with other lignocellulosic biomasses, such as eucalyptus and mango seed shell, which were 42% and 39%, respectively [27, 28]. Generally, the most effective XOS as a prebiotic for feed additives are those with degrees of polymerization less than or equal to 6. Hence, the abundance of these target XOS was analyzed for the hydrolysate from PM with increasing pretreatment temperature (Table 2). According to Table 2, the low-DP XOS yield was 10.5% at 170 °C, which means that 26.9% XOS (DP > 6) was formed. The XOS (DP > 6) content shows great potential for improving low-DP XOS production by enzymatic hydrolysis.

The results in Table 2 show that the yield of amino acids based on the original amino acid content increased with increasing reaction temperature. Considering that the optimal total XOS yield was obtained at 170 °C and that the optimal amino acid yield was 32.6% at 190 °C, which was only slightly higher than that at 170 °C (28.8%), the optimal temperature for amino acid and XOS production was 170 °C. Amino acids have been used in the diets of agricultural animals since the 1950s [29]. Hence, the amino acids found in the hydrolysate could be used as a byproduct and added to the diet of agricultural animals. The specific types and amounts of amino acids are listed in Table 3. Among the 17 amino acids in the autohydrolysate, 9 are essential amino acids for animals, including threonine, valine, methionine, isoleucine, leucine, phenylalanine, lysine, histidine and arginine, which together accounted for 45.9% of the total amino acid fraction. The amino acids recovered in the autohydrolysate showed the potential to be used as feed additives after purification.

### Optimization of the enzymatic hydrolysis of autohydrolysate

**Fig. 1:**
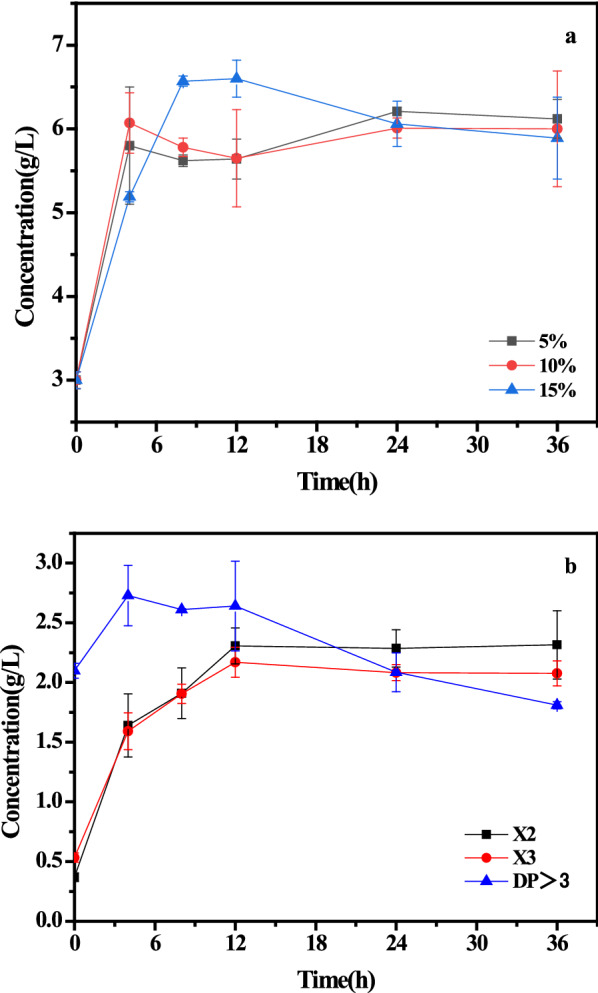
Time course for the production of low-DP XOS **a** from the enzymatic hydrolysis process with different enzyme dosages and xylobiose and xylotriose **b** from the enzymatic hydrolysis process with a 15% enzyme dosage

In vitro assays have proven that intestinal *bifidobacteria* prefer to metabolize xylobiose and xylotriose over high-DP XOS, with xylobiose usually undergoing preferential consumption from a DP-varying mixture of XOS [10]. According to the aforementioned results, the proportion of low-DP XOS in the XOS mixture obtained from autohydrolysis pretreatment was 27.7%, which was less than desired. To increase the yield of low-DP XOS, especially the yield of xylobiose and xylotriose, a strategy of mild enzymatic hydrolysis of the autohydrolysate was performed using endo-β-1–4-xylanase. Different enzyme dosages (5%, 10% and 15%) were added to hydrolyze the autohydrolysate (170 °C, 1 h) samples. The concentrations of low-DP XOS obtained from enzymatic hydrolysis are shown in Fig. 1.

It was shown that the concentration of low-DP XOS increased with increasing enzyme dosage, and the concentration of low-DP XOS increased from 3.1 g/L to 6.6 g/L at a substrate loading of 15% (v/v) within 12 h of enzymatic hydrolysis. Moreover, the concentrations of xylobiose and xylotriose stopped increasing at this substrate loading, maintaining 2.3 g/L and 2.2 g/L until the end of 36 h of enzymatic hydrolysis, respectively. However, the concentration of XOS (3 < DP ≤ 6) continued to decline after 12 h of enzymatic hydrolysis until the end of the process. The observed increase in xylobiose and xylotriose production occurred concurrently with the decline in XOS (DP > 3), illustrating that endo-β-1–4-xylanase can effectively cleave fragments of xylobiose and xylotriose from dissolved high-DP XOS. After 12 h of enzymatic hydrolysis, 68.7% of XOS was converted into low-DP XOS, which meant that the low-DP XOS yield was 25.8% based on the original xylan in the raw material. Overall, the concentration of low-DP XOS in the autohydrolysate was increased from 3.1 g/L to 6.6 g/L after enzymatic hydrolysis by endo-β-1–4-xylanase. Furthermore, the resulting amino acids in the autohydrolysate remained at the same concentration after enzymatic hydrolysis, which meant that endo-β-1–4-xylanase showed no effect on amino acids. Based on the aforementioned results, it can be seen that the low-DP XOS along with amino acids in the hydrolysate can be recovered after purification.

### Enzymatic hydrolysis of autohydrolyzed PM

**Fig. 2:**
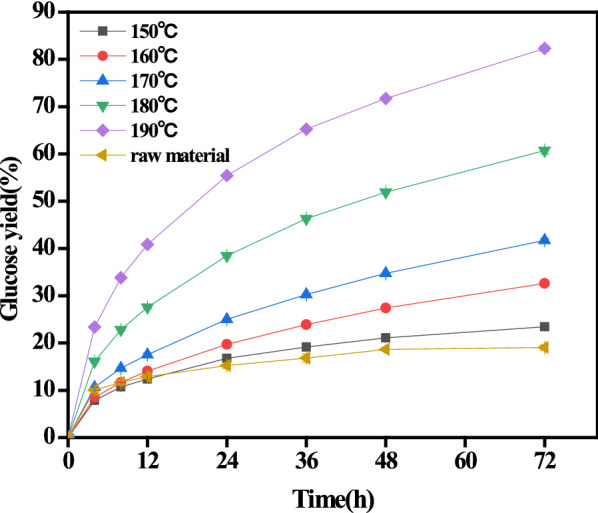
Effects of different temperatures on the glucose yield of autohydrolyzed PM

To explore the effects of reaction temperature on the subsequent enzymatic digestibility of the autohydrolyzed PM to produce glucose for SCP production, glucose yield (based on autohydrolyzed PM) was calculated for each enzymatic hydrolysis experiment and is shown in Fig. 2.

Figure 2 shows that autohydrolysis pretreatment with increasing reaction temperature showed positive effects on the enzymatic digestibility of glucan in the autohydrolyzed PM. Specifically, the enzymatic digestibility of glucan to glucose increased from 23.4 to 82.3% with increasing reaction temperature from 150 to 190 °C. The enhanced removal of xylan and lignin might be responsible for the increased enzymatic digestibility [11]. Moreover, a small amount of xylose in comparison with glucose was also found in the enzymatic hydrolysate (glucose:xylose = 4:1). Even though the highest glucose yield was obtained at 190 °C, when taking into consideration the desired XOS and amino acid yields, 170 °C was considered the optimal condition for the coproduction of these final products. Hence, the carbohydrates (glucose and xylose) in the enzymatic hydrolysate from PM autohydrolyzed at 170 °C are utilized as the carbon source in the further fermentation process.

### Optimization of SCP fermentation conditions

**Fig. 3:**
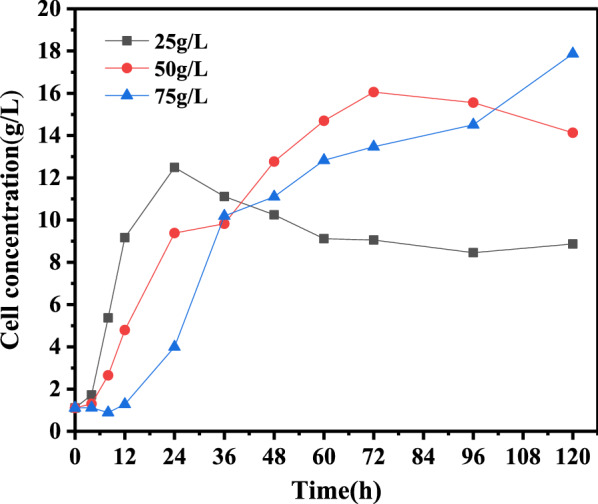
Biomass concentration during 120 h shake-flask cultivation with 25 g/L, 50 g/L and 75 g/L substrate concentrations of fermentable monosaccharides

**Fig. 4:**
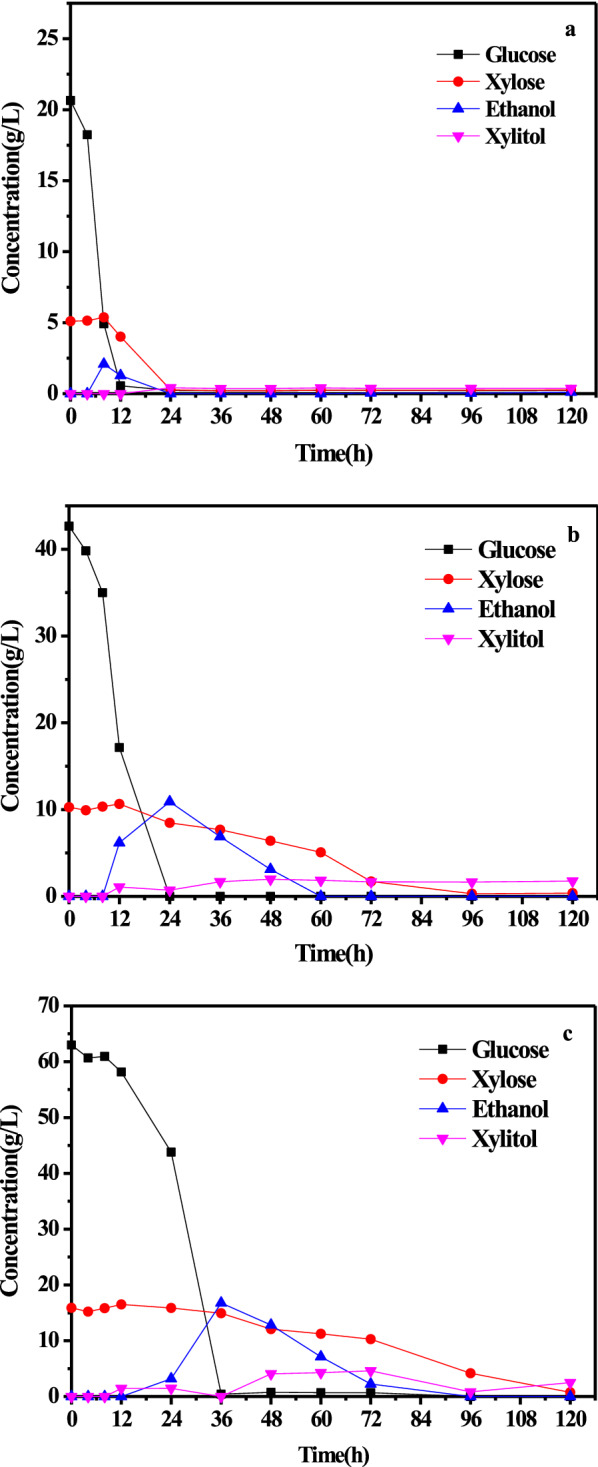
Consumption of fermentable monosaccharides and the accumulation of metabolites during 120 h shake-flask cultivation with 25 g/L (**a**), 50 g/L (**b**) and 75 g/L (**c**) substrate concentrations of fermentable monosaccharides

To investigate the influence of substrate concentration on the production of SCP, the enzymatic hydrolysate was evaporated to different fermentable monosaccharide (glucose and xylose) concentrations of 25 g/L, 50 g/L and 75 g/L. The enzymatic hydrolysate before and after evaporation was analyzed by HPLC and no inhibiting compounds (formic acid, acetic acid, levulinic acid, HMF and furfural) were found. The cell concentrations of SCP produced after fermentation with different substrates are shown in Fig. 3. The consumption of fermentable monosaccharides and the accumulation of metabolites are shown in Fig. 4.

As shown in Fig. 3, the cell concentration reached 12.5 g/L after 24 h of cultivation when the substrate concentration was 25 g/L. At this time, the glucose and xylose were both completely consumed. To increase the cell concentration, the fermentable monosaccharides (glucose and xylose) in the enzymatic hydrolysate were increased to 50 g/L and 75 g/L, which resulted in cell concentrations of 16.1 g/L and 17.9 g/L after 72 h and 120 h of cultivation, respectively. Under these fermentation processes, glucose and xylose were again both completely consumed. Hence, the most effective cultivation was observed when the fermentable monosaccharide (glucose and xylose) concentration was 75 g/L after 120 h of cultivation, indicating that the higher substrate concentration stimulates yeast accumulation. According to the consumption of fermentable monosaccharides and the accumulation of metabolites in Fig. 4, the glucose in 75 g/L enzymatic hydrolysate was totally consumed after 36 h of shake-flask cultivation, and the xylose was consumed after 120 h of cultivation. In addition, this phenomenon was verified when the concentration of fermentable monosaccharides in the enzymatic hydrolysate was 25 g/L and 50 g/L, which meant that *Candida utilis* utilized glucose in the substrate first and then utilized xylose in the substrate after the glucose was completely consumed. Overall, the optimal substrate concentration for SCP production was enzymatic hydrolysate (170 °C, 1 h) with 75 g/L fermentable monosaccharides (glucose and xylose).

### Mass balance

**Fig. 5:**
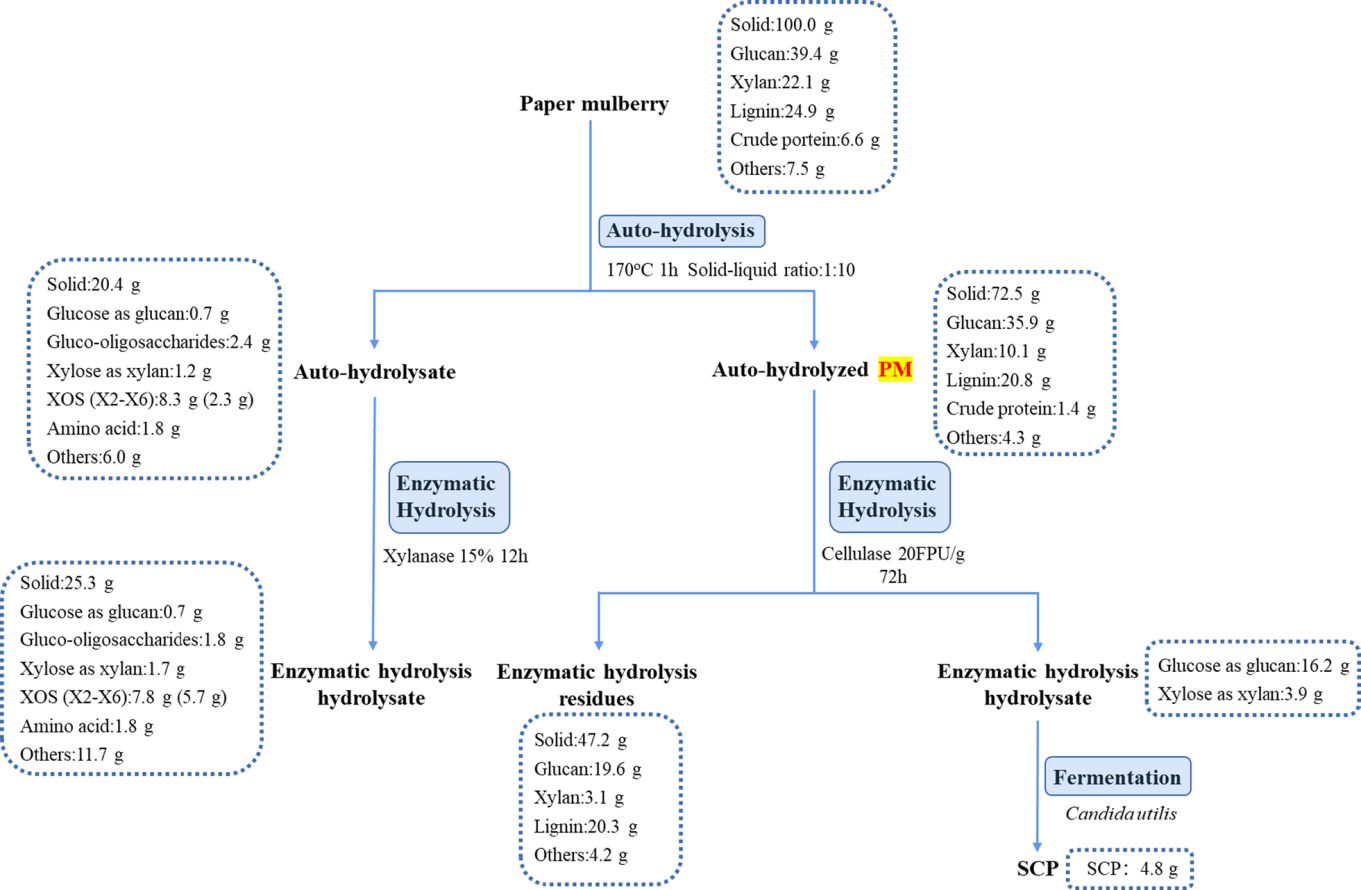
Mass balance for the production of amino acid-rich XOS and SCP from PM

To evaluate the feasibility and efficiency of the coproduction process, a mass balance (based on 100 g of dried PM) throughout the whole process is summarized in Fig. 5. Under the conditions of autohydrolysis at 170 °C for 1 h, 72.4 g of solid was recovered from 100 g of dried PM, including 35.9 g of glucan and 10.1 g of xylan. In the autohydrolysate, 8.3 g of XOS, including 2.3 g of low-DP XOS, and 1.8 g of amino acids could be recovered. The total XOS yield and low-DP XOS yield were 37.4% and 10.5%, respectively. Moreover, 28.8% of the original protein was recovered in the autohydrolysate in the form of amino acids. After 12 h of enzymatic hydrolysis of the autohydrolysate with endo-β-1–4-xylanase (15% enzyme dosage), 5.7 g of low-DP XOS (25.8%) and 1.8 g of amino acids (28.6%) were obtained. Then, by enzymatic hydrolysis of the autohydrolyzed PM (170 °C, 1 h), 20.1 g of fermentable monosaccharides (16.2 g of glucose and 3.9 g of xylose) could be obtained for fermentation to produce 4.8 g of SCP. These results indicate that the branches and twigs of PM show good performance in XOS and fermentable monosaccharide production. Chen et al. reported that 14.4 g of XOS and 30.4 g of glucose could be recovered from 100 g of reed scraps after autohydrolysis at 170 °C for 30 min [30]. Neto et al. reported the optimization of XOS production through the hydrothermal pretreatment of *Eucalyptus *[27]. The sawdust from *Eucalyptus* shows the potential for the production of XOS, containing 60 mg of XOS per gram of *Eucalyptus*. However, no amino acids were reported to be found in autohydrolysate from these reported lignocellulosic biomasses. Due to its high protein content, PM is an excellent raw material to coproduce the amino acid-rich XOS and SCP through autohydrolysis, enzymatic hydrolysis, and fermentation.

## Conclusion

In this study, a new process was proposed to obtain amino acids, low-DP XOS and SCP from PM through autohydrolysis. After autohydrolysis (170 °C, 1 h) and enzymatic hydrolysis (15% enzymatic dosage), 6.6 g/L low-DP XOS and 2.4 g/L amino acids were produced from autohydrolysate. Autohydrolyzed PM was used to produce fermentation monosaccharides for SCP, and 17.9 g/L SCP was obtained from enzymatic hydrolysate with 75 g/L fermentable monosaccharides. These products, including amino acid-rich XOS and SCP showed the potential to make progress toward the industrialization of a composite feed additive as a high-value application based on low-cost autohydrolysis.

## Methods

### Materials and reagents

The branches and twigs of PM were harvested from the planting at the Guangzhou Academy of Forestry. After air-drying for 24 h, the branches and twigs were ground and sieved through a 40-mesh sieve. The resultant PM sawdust was stored at room temperature until processing. The crude endo-β-1–4-xylanase used in this study was produced from *Trichoderma reesei* and provided by Jiangsu Kangwei Biotechnology Co., Ltd. of China. The measured enzyme activity was 30 U/mL. Commercial cellulase (Cellic® CTec.2.0) was provided by Novozymes North America (Franklinton, NC). Bradford reagent and bovine serum protein standard solution were purchased from Sigma–Aldrich (Shanghai, China). Other chemicals, such as H_2_SO_4_, MgSO_4_, CaCl_2_, KH_2_PO_4_ and (NH_4_)_2_SO_4_, were of analytical grade and were purchased from Nanjing Reagent Company (Nanjing, China).

### Autohydrolysis of PM

For autohydrolysis pretreatment, 7 g of PM sawdust (based on dry weight) was pretreated in a sealed 100 mL reactor held in an oil bath at 150 °C, 160 °C, 170 °C, 180 °C or 190 °C for 1 h. The heat-up time was 30 min. An appropriate volume of deionized water was added to the vessel to set the final solid-to-liquid ratio at 1:10. After pretreatment, the reactors were cooled by submersion in an ice water bath for 15 min. The solid and liquid fractions were separated by vacuum filtration, and the solid fraction was washed with distilled water until the pH of the filtrate reached 7.0. The washed solid fraction and liquid fraction were refrigerated at 4 °C for the following experiments. The recovery yields of solids and glucan, the removal yields of xylan and lignin and the degradation yield of protein were calculated using the following formulas.

1 $$ \begin{aligned} & {\text{Recovery}}\, {\text{yield}} \,{\text{of}}\, {\text{Solid}}\left( \% \right) \\ &\quad= \frac{{{\text{Mass}}\, {\text{of}}\, {\text{autohydrolyzed}} \,{\text{PM}}}}{{{\text{Mass}} \,{\text{of}}\, {\text{raw}}\, {\text{PM}}}} \times 100\% \end{aligned} $$

2 $$ \begin{aligned} & {\text{Recovery}} \,{\text{yield}}\, {\text{of}} \,{\text{Glucan}}\left( \% \right) \\ &\quad= \frac{{{\text{Glucan}} \,{\text{in}}\, {\text{autohydrolyzed}}\, {\text{PM}}}}{{{\text{Glucan}}\, {\text{in}} \,{\text{raw PM}}}} \times 100\% \end{aligned} $$

3 $$ \begin{aligned} & {\text{Removal}}\, {\text{yield}}\, {\text{of}}\, {\text{Xylan}}\left( \% \right) \\ &\quad= 1 - \frac{{{\text{Xylan}}\, {\text{in}} \,{\text{autohydrolyzed}} \,{\text{PM}}}}{{{\text{Xylan}} \,{\text{in}}\, {\text{raw}}\, {\text{PM}}}} \times 100\% \end{aligned} $$

4 $$ {\text{Removal}}\, {\text{yield}} \,{\text{of}} \,{\text{Lignin}}\left( \% \right) = 1 - \frac{{{\text{Lignin}}\, ({\text{acid}} - {\text{insoluble}} + {\text{acid}} - {\text{soluble}})\, {\text{in}}\, {\text{autohydrolyzed}}\, {\text{PM}}}}{{{\text{Lignin}} ({\text{acid}} - {\text{insoluble}} + {\text{acid}} - {\text{soluble}})\, {\text{in}}\, {\text{raw}}\, {\text{PM}}}} \times 100\% $$

5 $$ \begin{aligned} & {\text{Degradation}}\, {\text{yield}} \,{\text{of}}\, {\text{Protein}}\left( \% \right) \\ &\quad= 1 - \frac{{{\text{Protein}}\, {\text{in}} \,{\text{autohydrolyzed}} \,{\text{PM}}}}{{{\text{Protein}} \,{\text{in}} \,{\text{raw}} \,{\text{PM}}}} \times 100\% . \end{aligned} $$

### Enzymatic hydrolysis of autohydrolysate

Before enzymatic hydrolysis, the autohydrolysate was first centrifuged, and filtered to remove suspended solid fractions. Next, the pH of the autohydrolysate was adjusted using 1 M citrate buffer to reach ~ 4.8. Ten milliliter samples with adjusted pH values were mixed with endo-β-1–4-xylanase at substrate loadings of 5%, 10%, and 15% (v/v). The enzymatic hydrolysis assays were performed in a constant temperature oscillator (50 °C, 150 rpm) for 36 h. Aliquots (0.5 mL) were taken at 4 h, 8 h, 12 h, 24 h and 36 h for HPAEC (high-performance anion exchange chromatography) analysis of the amount of low-DP XOS.

### Enzymatic hydrolysis of autohydrolyzed PM

Enzymatic hydrolysis of the autohydrolyzed PM was carried out by suspending the pretreated solid biomass in sodium citrate buffer (pH 4.8) at 2% glucan content (w/v) with an enzyme loading of 20 FPU/g glucan. The suspensions were incubated at 50 °C and 150 rpm for 72 h. Aliquots (0.6 mL) were taken at 4 h, 8 h, 12 h, 24 h, 36 h, 48 h, 60 h and 72 h to determine the sugar concentration by HPLC to calculate the glucose yields as follows:

$$ {\text{ Glucose}}\, {\text{yield}}\% = \frac{{{\text{Glucose}}\, {\text{as}}\, {\text{glucan}} \,{\text{in}}\, {\text{enzymatic}} \,{\text{hydrolysate}}\;({\text{g}})}}{{{\text{initial}}\, {\text{Glucan}}\, {\text{in}} {\text{substrate}}\,({\text{g}})}} \times 100\% . $$

### Fermentation to produce SCP

To obtain specified substrate concentrations (25 g/L, 50 g/L and 75 g/L fermentable monosaccharides: glucose and xylose) from the enzymatic hydrolysate, the liquid fraction was evaporated to a particular volume by rotary evaporation. Various nutritive salts were then added to assist with fermentation, including 0.6 g/L MgSO_4_, 0.13 g/L CaCl_2_, 6 g/L KH_2_PO_4_, and 5 g/L N sources. The nitrogen sources were (NH_4_)_2_SO_4_ and dried corn steep liquor powder at a ratio of 2:1. The yeast strain for fermentation was *Candida utilis*. This experiment was carried out in 250 mL flasks with a working volume of 50 mL at 30 °C and 170 rpm for 120 h. Aliquots (1.0 mL) were taken at 4 h, 8 h, 12 h, 24 h, 36 h, 48 h, 60 h, 72 h, 96 h and 120 h for further analysis.

### Analysis methods

#### Chemical compositional analysis of raw and autohydrolyzed PM

The chemical compositions of raw PM and autohydrolyzed PM were analyzed according to the standard of the National Renewable Energy Laboratory (NREL) for analyzing biomass [31]. First, 0.3 g of biomass (40 mesh) was hydrolyzed with 72% H_2_SO_4_ for 1 h. The hydrolyzed biomass was diluted to 4% H_2_SO_4_ and autoclaved at 121 °C for 1 h. The hydrolysate was filtered and oven-dried to determine the insoluble solids, and the filtrate was collected for the determination of acid-soluble lignin (UV–Vis at 205 nm and 110 Abs coefficient) and monosaccharides by HPLC (high-performance liquid chromatography) equipped with an Aminex Bio–Rad HPX-87H column and a refractive index detector. Sugar analysis was performed with 0.05 M H_2_SO_4_ solution as the mobile phase flowing at a rate of 0.6 mL/min and a column temperature of 50 °C. The content of acid-insoluble lignin was determined by the weighting method. Moreover, the crude protein was determined by using the Kjeldahl method described by AOAC [32].

#### The chemical compositional analysis of autohydrolysate

An aliquot of autohydrolysate was hydrolyzed using 4% sulfuric acid at 121 °C for 1 h to convert xylooligosaccharides into xylose. Aliquots of autohydrolysate before and after acid hydrolysis were analyzed by the same aforementioned HPLC system. The concentration of total XOS was measured by the differences in the respective xylose contents before and after acid hydrolysis. The concentration of low-DP XOS in the autohydrolysate was determined by HPAEC (Dionex ICS-3000, Thermo Fisher Scientific, Waltham, MA, USA) with a CarboPac PA 200 column (Thermo Fisher Scientific, Waltham, MA, USA) at 30 °C. Eluents of 0.1 M NaOH and 0.5 M NaOAc (sodium acetate) containing 0.1 M NaOH were used at a gradient elution of 0.3 mL/min. Low-DP XOS was detected using a Dionex ED 40 Electrochemical Detector in pulsed amperometry mode through the standard quadruple waveform. Additionally, an HPLC system equipped with an AccQ Tag amino acid analysis column and photodiode array detector was used to determine the types and concentrations of amino acids. The analysis was performed with ultrapure water, 60% (v/v) acetonitrile solution as the mobile phase flowing at a rate of 1 mL/min, and a column temperature of 35 °C. The concentration of protein in the autohydrolysate was quantified by Bradford assay [33]. The cell concentration was determined by spectrophotometry, where the optical density of the cell suspension was measured against a standard curve (absorbance at 600 nm). The conversion of UV reading to cell biomass in g/L follows the standard curve (y = 0.38 × − 0.03, R^2^ = 0.9909). The concentrations of fermentable monosaccharides and metabolites in the supernatant were analyzed by the aforementioned HPLC system.

## Data Availability

All data generated and analyzed in this study are included in this published article.

## Abbreviations

XOS: Xylooligosaccharide
low-DP XOS: XOS with low degrees of polymerization (DP ≤ 6)
SCP: Single-cell protein
NREL: National Renewable Energy Laboratory
HPLC: High-performance liquid chromatography
HPAEC: High-performance anion exchange chromatography

## References

[1] Peng X, Liu H, Chen P, Tang F, Hu Y, Wang F (2019). A Chromosome-scale genome assembly of paper mulberry (Broussonetia papyrifera) provides new insights into its forage and papermaking usage. Mol Plant.

[2] Han Q, Wu Z, Huang B, Sun L, Ding C, Yuan S (2016). Extraction, antioxidant and antibacterial activities of Broussonetia papyrifera fruits polysaccharides. Int J Biol Macromol.

[3] Tian JL, Liu TL, Xue JJ, Hong W, Zhang Y, Zhang DX (2019). Flavanoids derivatives from the root bark of Broussonetia papyrifera as a tyrosinase inhibitor. Ind Crops Prod.

[4] Malaník M, Treml J, Leláková V, Nykodýmová D, Oravec M, Marek J (2020). Anti-inflammatory and antioxidant properties of chemical constituents of Broussonetia papyrifera. Bioorg Chem.

[5] Ryu HW, Curtis-Long MJ, Jung S, Jeong IY, Kim DS, Kang KY (2012). Anticholinesterase potential of flavonols from paper mulberry (Broussonetia papyrifera) and their kinetic studies. Food Chem.

[6] Zhang Q, Li M, Luo B, Luo Y, Jiang H, Chen C (2021). In situ growth gold nanoparticles in three-dimensional sugarcane membrane for flow catalytical and antibacterial application. J Hazard Mater.

[7] Huang C, Fang G, Zhou Y, Du X, Yu L, Meng X (2020). Increasing the carbohydrate output of bamboo using a combinatorial pretreatment. ACS Sustain Chem Eng..

[8] Galia A, Schiavo B, Antonetti C, Galletti AMR, Interrante L, Lessi M (2015). Autohydrolysis pretreatment of Arundo donax: a comparison between microwave-assisted batch and fast heating rate flow-through reaction systems. Biotechnol Biofuels.

[9] Otieno DO, Ahring BK (2012). A thermochemical pretreatment process to produce xylooligosaccharides (XOS), arabinooligosaccharides (AOS) and mannooligosaccharides (MOS) from lignocellulosic biomasses. Bioresour Technol.

[10] Gullón P, Salazar N, Muñoz MJG, Gueimonde M, Ruas-Madiedo P, De los Reyes-Gavilán CG (2011). Assessment on the fermentability of xylooligosaccharides from rice husks. BioResources.

[11] Huang C, Jeuck B, Du J, Yong Q, Chang HM, Jameel H (2016). Novel process for the coproduction of xylo-oligosaccharides, fermentable sugars, and lignosulfonates from hardwood. Bioresour Technol.

[12] Sutton TA, O’Neill HVM, Bedford MR, McDermott K, Miller HM (2021). Effect of xylanase and xylo-oligosaccharide supplementation on growth performance and faecal bacterial community composition in growing pigs. Anim Feed Sci Technol.

[13] He X, Yu B, He J, Huang Z, Mao X, Zheng P (2020). Effects of xylanase on growth performance, nutrients digestibility and intestinal health in weaned piglets. Livest Sci.

[14] Schwab CG (2011). Feed supplements : ruminally protected amino acids amino acid nutrition of dairy cattle.

[15] Sharif M, Zafar MH, Aqib AI, Saeed M, Farag MR, Alagawany M (2021). Single cell protein: Sources, mechanism of production, nutritional value and its uses in aquaculture nutrition. Aquaculture.

[16] Ritala A, Häkkinen ST, Toivari M, Wiebe MG (2017). Single cell protein-state-of-the-art, industrial landscape and patents 2001–2016. Front Microbiol.

[17] Jones SW, Karpol A, Friedman S, Maru BT, Tracy BP (2020). Recent advances in single cell protein use as a feed ingredient in aquaculture. Curr Opin Biotechnol.

[18] Hatoum R, Labrie S, Fliss I (2012). Antimicrobial and probiotic properties of yeasts: from fundamental to novel applications. Front Microbiol.

[19] Ezekiel O, Aworh O (2018). Simultaneous saccharification and cultivation of Candida utilis on cassava peel. Innov Food Sci Emerg Technol.

[20] Buitrago Mora HM, Piñeros MA, Espinosa Moreno D, Restrepo Restrepo S, Cardona Jaramillo JEC, Álvarez Solano A (2019). Multiscale design of a dairy beverage model composed of Candida utilis single cell protein supplemented with oleic acid. J Dairy Sci.

[21] dos Santos JF, Canettieri EV, Souza SMA, Rodrigues RCLB, Martínez EA (2019). Treatment of sugarcane vinasse from cachaça production for the obtainment of Candida utilis CCT 3469 biomass. Biochem Eng J.

[22] Kim DS, Myint AA, Lee HW, Yoon J, Lee YW (2013). Evaluation of hot compressed water pretreatment and enzymatic saccharification of tulip tree sawdust using severity factors. Bioresour Technol.

[23] Narron RH, Kim H, Chang HM, Jameel H, Park S (2016). Biomass pretreatments capable of enabling lignin valorization in a biorefinery process. Curr Opin Biotechnol.

[24] He J, Huang C, Lai C, Huang C, Li M, Pu Y (2020). The effect of lignin degradation products on the generation of pseudo-lignin during dilute acid pretreatment. Ind Crops Prod.

[25] Sun SN, Li HY, Cao XF, Xu F, Sun RC (2015). Structural variation of eucalyptus lignin in a combination of hydrothermal and alkali treatments. Bioresour Technol.

[26] Batalha LAR, Han Q, Jameel H, Chang HM, Colodette JL, BorgesGomes FJ (2015). Production of fermentable sugars from sugarcane bagasse by enzymatic hydrolysis after autohydrolysis and mechanical refining. Bioresour Technol.

[27] Neto FSPP, Roldán IUM, Galán JPM, Monti R, de Oliveira SC, Masarin F (2020). Model-based optimization of xylooligosaccharides production by hydrothermal pretreatment of Eucalyptus by-product. Ind Crops Prod.

[28] Monteiro CRM, Ávila PF, Pereira MAF, Pereira GN, Bordignon SE, Zanella E (2021). Hydrothermal treatment on depolymerization of hemicellulose of mango seed shell for the production of xylooligosaccharides. Carbohydr Polym.

[29] Kidd MT, Tillman PB, Waldroup PW, Holder W (2013). Feed-grade amino acid use in the United States: The synergetic inclusion history with linear programming. J Appl Poult Res.

[30] Chen M, Lu J, Cheng Y, Li Q, Wang H (2019). Novel process for the coproduction of xylo-oligosaccharide and glucose from reed scraps of reed pulp mill. Carbohydr Polym.

[31] Sluiter A, Hames B, Ruiz R, Scarlata C, Sluiter J, Templeton D, et al. Determination of structural carbohydrates and lignin in biomass: laboratory analytical procedure (LAP); Issue Date: April 2008; Revision Date: July 2011 (Version 07-08-2011). 2011;2011.

[32] AOAC (2005). Official methods of analysis of AOAC International.

[33] Bradford MM (1976). A rapid and sensitive method for the quantitation of microgram quantities of protein utilizing the principle of protein-dye binding. Anal Biochem.

